# Respiration activity monitoring system for any individual well of a 48-well microtiter plate

**DOI:** 10.1186/s13036-016-0034-3

**Published:** 2016-10-27

**Authors:** David Flitsch, Sebastian Krabbe, Tobias Ladner, Mario Beckers, Jana Schilling, Stefan Mahr, Uwe Conrath, Werner K. Schomburg, Jochen Büchs

**Affiliations:** 1 AVT - Aachener Verfahrenstechnik, Biochemical Engineering, RWTH Aachen University, Worringer Weg 1, 52074 Aachen, Germany; 2 KEμ, Konstruktion und Entwicklung von Mikrosystemen, RWTH Aachen University, Steinbachstraße 53b, 52074 Aachen, Germany; 3 Institute of Plant Physiology, Aachen Biology and Biotechnology, RWTH Aachen University, 1 Worringer Weg, Aachen, 52074 Germany

**Keywords:** μRAMOS, RAMOS, High throughput, Microtiter plate (MTP), Oxygen transfer rate (OTR)

## Abstract

**Background:**

Small-scale micro-bioreactors have become the cultivation vessel of choice during the first steps of bioprocess development. They combine high cultivation throughput with enhanced cost efficiency per cultivation. To gain the most possible information in the early phases of process development, online monitoring of important process parameters is highly advantageous. One of these important process parameters is the oxygen transfer rate (OTR). Measurement of the OTR, however, is only available for small-scale fermentations in shake flasks via the established RAMOS technology until now. A microtiter plate-based (MTP) μRAMOS device would enable significantly increased cultivation throughput and reduced resource consumption. Still, the requirements of miniaturization for valve and sensor solutions have prevented this transfer so far. This study reports the successful transfer of the established RAMOS technology from shake flasks to 48-well microtiter plates. The introduced μRAMOS device was validated by means of one bacterial, one plant cell suspension culture and two yeast cultures.

**Results:**

A technical solution for the required miniaturized valve and sensor implementation for an MTP-based μRAMOS device is presented. A microfluidic cover contains in total 96 pneumatic valves and 48 optical fibers, providing two valves and one optical fiber for each well. To reduce costs, an optical multiplexer for eight oxygen measuring instruments and 48 optical fibers is introduced. This configuration still provides a reasonable number of measurements per time and well. The well-to-well deviation is investigated by 48 identical *Escherichia coli* cultivations showing standard deviations comparable to those of the shake flask RAMOS system. The yeast *Hansenula polymorpha* and parsley suspension culture were also investigated.

**Conclusions:**

The introduced MTP-based μRAMOS device enables a sound and well resolved OTR monitoring for fast- and slow-growing organisms. It offers a quality similar to standard RAMOS in OTR determination combined with an easier handling. The experimental throughput is increased 6-fold and the media consumption per cultivation is decreased roughly 12.5-fold compared to the established eight shake flask RAMOS device.

**Electronic supplementary material:**

The online version of this article (doi:10.1186/s13036-016-0034-3) contains supplementary material, which is available to authorized users.

## Background

For the first steps of bioprocess development, small-scale micro-bioreactors are progressively replacing the classical shake flask as the cultivation vessel of choice [1–3]. The main reason for this is the higher cultivation throughput combined with enhanced cost efficiency [4, 5]. To gain high information content even in early process development phases, online monitoring of important process parameters is essential. Therefore, in the recent past, powerful micro-bioreactor systems such as the BioLector [6, 7], the μ24 system [8] or stirred ambr bioreactors [9, 10] with online monitoring capabilities were developed and introduced to the market. However, the mentioned microtiter plate-based systems (BioLector and μ24 system) are not able to measure the important process parameter oxygen transfer rate (OTR) in any individual well [11]. The OTR provides important information about growth behavior and metabolism, it enables mass balancing and evaluation of stoichiometric relationships for aerobic cultivations [12–15]. Even estimations about the product formation are possible on the basis of the OTR [16, 17]. According to the state of the art, the OTR is determined via off gas analytics in fermenter scale [18]. For small-scale cultivations in shake flasks, the OTR is accessible by means of the RAMOS technology. RAMOS stands for respiration activity monitoring system and was introduced 2001 by Anderlei et al. [19, 20]. The RAMOS technology requires the ability to switch repeatedly between times of a sealed and a properly gassed bioreactor. During the sealed and so called stop phases the oxygen partial pressure, measured via a respective sensor, decreases due to respiration of the cultivated microorganisms or cells. The OTR from the gas to the aqueous phase is proportional to the determined slope of the oxygen partial pressure during stop phases [19, 20]. In summary, the RAMOS technology requires primarily two valves and one oxygen sensor per cultivation vessel to determine the oxygen transfer rate. Due to the compact dimensions of MTPs, integration of the required technical components for each well is challenging and has so far prevented the transfer of the RAMOS technology from shake flask to the MTP scale. The aim of this study is the development of a suitable valve and sensor technology to realize a micro-bioreactor μRAMOS system, based on a standard 48-well MTP. This would lead to an easier handling and a 6-fold increased cultivation throughput combined with strongly reduced resource consumption per cultivation.

## Results and discussion

### Developed measurement setup

**Fig. 1:**
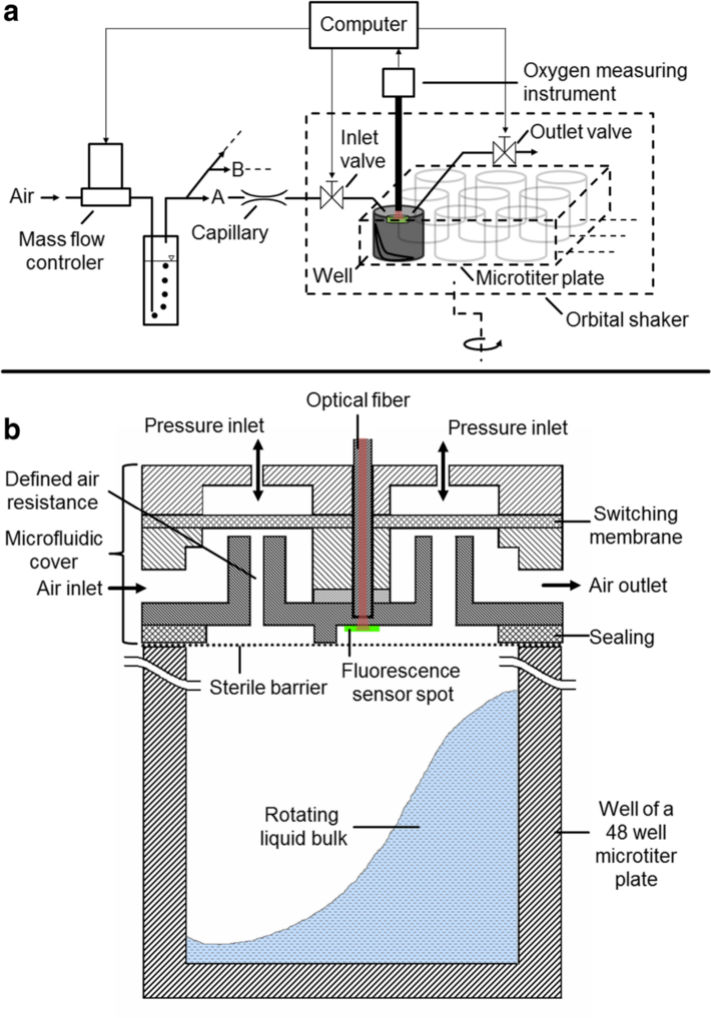
**a** Schematic overview of the developed μRAMOS system for measuring the oxygen transfer rate in each single well. **b** Single well schematic cross section of the microfluidic MTP cover for OTR measurements. Valves, oxygen sensors, optical fibers, oxygen measuring instruments and capillaries are only shown for one well. All valves as well as the mass flow controller are controlled by a computer. The oxygen partial pressures of the headspaces of every well are detected via oxygen measuring instruments and monitored and analyzed by the computer. The MTP is mounted on an orbital shaker. The capillaries ensure an equal air flow through every well of the MTP. By applying overpressure through the pressure inlets, the elastic switching membrane seals the air inlet and outlet to realize an interruption of the air flow during the cultivation. The fluorescence sensor spot is glued onto the lower side of the cover facing the well headspace. The optical fiber is connected to an oxygen measuring instrument via an optical multiplexer on one end and plugged in the microfluidic cover facing the fluorescence sensor spot on the other end. Due to the transparent material, the oxygen dependent fluorescence behavior of the immobilized fluorescence spot can be excited and non-invasively detected through the material

The schematic overview of the newly developed μRAMOS system for measuring the oxygen transfer rate in individual wells of a 48-well MTP is shown in Fig. 1a. As mentioned above, the RAMOS principle requires the ability to switch repeatedly between an active gassing phase (flow phase) and a sealed phase (stop phase) of the bioreactor during cultivations while (semi-) continuously measuring the oxygen partial pressure [19, 20]. During the sealed stop phases the oxygen transfer rate is given by Eq. 1:

1 $$ \boldsymbol{O}\boldsymbol{T}\boldsymbol{R} = \frac{\varDelta {\boldsymbol{p}}_{{\boldsymbol{O}}_2}}{\varDelta \boldsymbol{t}}\cdotp \frac{{\boldsymbol{V}}_{\boldsymbol{G}}}{\boldsymbol{R}\cdotp \boldsymbol{T}\cdotp {\boldsymbol{V}}_{\boldsymbol{L}}} $$

where *OTR* is the oxygen transfer rate [mol L^-1^ h^-1^], $\frac{\varDelta {\boldsymbol{p}}_{{\boldsymbol{O}}_2}}{\varDelta \boldsymbol{t}}$ is the oxygen partial pressure change over time during stop phases [Pa h^-1^], *V*_*G*_ is the gas volume of the sealed well during stop phases [L], *R* is the gas constant [Pa mol^-1^ K^-1^], *T* is the temperature [K] and *V*_*L*_ is the liquid volume of the cultivation broth [L]. Neglecting evaporation of the cultivation broth and temperature changes, the oxygen transfer rate is determined by the slope of the measured oxygen partial pressure over time during stop phases. To switch between flow and stop phases, the air supply is controlled by one inlet and one outlet valve per well and defined by a single mass flow controller (GF40, Brooks Instrument GmbH, Dresden, Germany) per MTP (Fig. 1a). Due to the equal air resistance of 48 identical capillaries, an equal air supply of the single wells is accomplished. The MTP (MTP-R48-B, m2p-labs GmbH, Baesweiler, Germany) is fixed on an in house built orbital shaker (shaking diameter *d*_*0*_ = 3 mm). The valves and the mass flow controller are controlled by a computer.

Only optical approaches fulfill the requirement of a minimal space demand to measure the oxygen partial pressure. It is known that the luminescence of specific fluorescence dyes can be so called “quenched” due to the presence of oxygen [21], meaning that emission of the fluorescence photon is prevented by a prior energy transfer to an oxygen molecule after collision. In this way, the oxygen partial pressure is linked to the luminosity of the fluorescence dye via the Stern-Vollmer equation [22]. Accordingly, the fluorescence lifetime is influenced and can be utilized as a measurand in oxygen analytics [23]. An elegant method to determine the fluorescence lifetime is the detection of the time shift (often called phase shift) between an intensity modulated excitation and a consequently modulated fluorescence light detection via an integrated lock-in amplifier [24]. In this way, the demand for high-end electronics with high temporal resolutions can be avoided. The commercially available and utilized optical oxygen measuring instruments (Piccolo2-OEM, Pyro Science GmbH, Aachen, Germany) work with an intensity modulation frequency in the kHz range. The applied fluorescence dye is immobilized in fluorescence sensor spots (OXSP5, Pyro Science GmbH, Aachen, Germany) [25, 26]. The red light excitation and infrared light emission is advantageous for avoiding biogenic fluorescences [27, 28]. For well-resolved oxygen partial pressure measurements during stop phases, every well is equipped with a respective optical fiber and a fluorescence sensor spot (Fig. 1b).

Due to the limited space, commercially available valves turned out to be unsuitable to provide two valves for every well. Figure 1b shows a schematic single-well cross-section of the developed microfluidic MTP cover [29]. This cover is located on top of the MTP, which is covered by a gas permeable sterile barrier (900371-T, HJ-Bioanalytik GmbH, Erkelenz, Germany). Within this microfluidic MTP cover, two valves, one defined air resistance and one optical fiber are included for every well. Additionally, the cover contains a combined channel system to merge all air inlets of the 48 wells (not shown in Fig. 1b). In this way, only one air supply tube has to be connected to the cover. To close the inlet or outlet valve, a molded elastic silicone switching membrane (Elastosil RT 625, Wacker Chemie AG, Munich, Germany) is located above the respective valve seat. By applying over or under pressure above the switching membrane, the air inlet of the valve seat is sealed or properly opened. All inlet valves are connected to the same pressure channel system within the microfluidic MTP cover and consequently switch simultaneously. An analogue channel system for the outlet valves is integrated equally. To ensure a homogenous air supply throughout all wells, the valve seat diameter of every inlet valve is sufficiently small (diameter: 200 μm) to generate a high air resistance (depicted in Fig. 1a as capillary). To non-invasively measure the oxygen partial pressure of the gas in the headspace of the wells, the material of the microfluidic MTP cover (Acrylglas XT (PMMA), FAKU GmbH, Cologne, Germany) is transparent for the red excitation and near infrared fluorescence light.

**Fig. 2:**
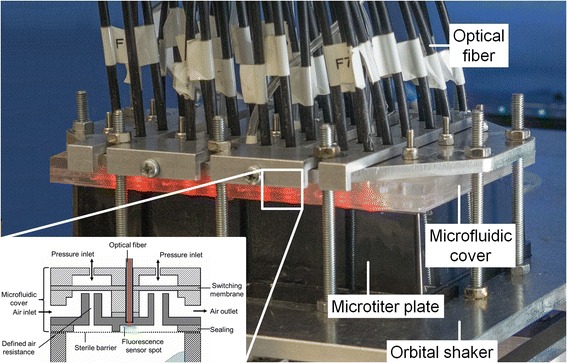
Picture of the applied μRAMOS system for measuring the oxygen transfer rate in each single well. Single well schematic cross section of the microfluidic MTP cover for OTR measurements is according to Fig. 1B. The microtiter plate is mounted on an orbital shaker. The fluorescence sensor spot is glued onto the lower side of the cover facing the well headspace and excited via red light. The optical fiber is connected to an optical multiplexer on one end (Fig. 4) and plugged in the microfluidic cover facing the fluorescence sensor spot on the other end

Figure 2 shows the applied μRAMOS setup with respective microfluidic cover. The 48-well MTP is located on the in-house built orbital shaker. The developed microfluidic cover is located on top of the MTP and contains 48 optical fibers. With the help of an optical multiplexer, the oxygen partial pressures of every row (eight wells) of the MTP are measured sequentially (see detailed description below). Due to the transparent material PMMA, the red excitation light, for the simultaneous oxygen partial pressure measurement within one row (eight wells), is visible in Fig. 2.

**Fig. 3:**
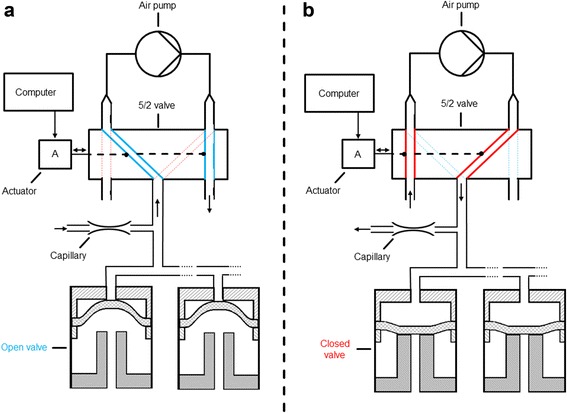
Illustration of the switching mechanism of the developed μRAMOS technology. **a** Switch position of the 5/2 valve for providing a under pressure and (**b**) over pressure above the switching membrane. Two sets of air pumps, capillaries and 5/2 valves are used for switching all 48 inlet valves independently of the 48 outlet valves. Differential switching pressures: - 300 hPa ((**a**) open valve (*blue*)), + 600 hPa ((**b**) closed valve (*red*)). The pressures are adjusted by the air resistances of the capillaries and the air pump characteristics

Figure 3 shows an illustration of the switching mechanism and the implementation of the over and under pressure supply. The necessary pressures are generated by a constantly operating air pump (7 s57012, Schwarzer Precision GmbH + Co. KG, Essen, Germany). The 5/2 valve (combination of three 3/2 valves, 00288231, Bürkert GmbH, Ingelfingen, Germany) is controlled by a computer and enables instant switching between the under and over pressure port of the applied air pump. The utilized dynamic pressures are defined by the performance of the air pump and the air resistance of the integrated capillary (diameter: 0.25 mm, length: 20 mm). By operating at dynamic pressures instead of maximal pressures with no air flow, the lifetime of the air pump is highly prolonged. Two sets of air pumps, 5/2 valves and capillaries are applied to switch all 48 inlet valves independently from the 48 outlet valves.

**Fig. 4:**
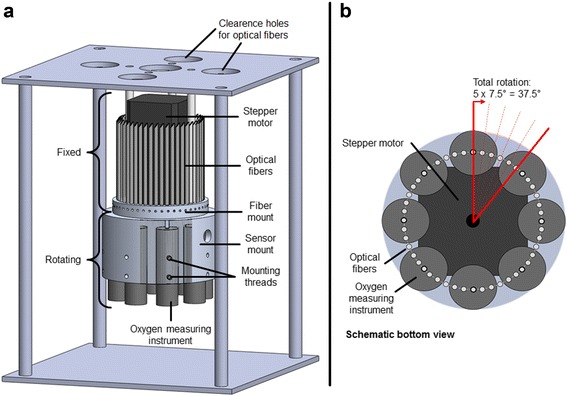
CAD drawing side view (**a**) and schematic bottom view (**b**) of the optical multiplexer (8 × 48) for measurements of the oxygen partial pressure in each well. Eight oxygen measuring instruments are rotated against 48 fixed optical fibers. The rotation sequence for oxygen partial pressure measurement through all 48 fibers consists of five 7.5° steps and a reversed – 37.5° step. Complete cycle time is roughly 3.5 s. Only the first 5 cm of the optical fibers are shown in (a) for clarity

To allow for sufficient measurement rates and a reasonable number of oxygen instruments (Piccolo2-OEM, Pyro Science GmbH, Aachen, Germany) an optical multiplexer was developed. Figure 4a shows the in house built optical multiplexer (8 × 48) for measuring the oxygen partial pressure in each well. With the help of a stepper motor (ZAB-T-NM17A04-KT03, Laser 2000 GmbH, Wessling, Germany), eight oxygen measuring instruments are rotated against 48 fixed optical fibers (modified PICFIB2, Pyro Science GmbH, Aachen, Germany). The rotation sequence for oxygen partial pressure measurement through all 48 fibers consists of five 7.5° steps and a reversed – 37.5° step (Fig. 4b). Hence, an oxygen partial pressure measurement can be performed every 3.5 s in each well, resulting in a quasi-continuous signal. The developed measurement software is LabVIEW based (LabVIEW 12, National Instruments Germany GmbH, Munich, Germany), since LabVIEW drivers were provided by the manufacturers of the oxygen measuring instruments and stepper motor (Pyro Science GmbH, Laser 2000 GmbH).

**Fig. 5:**
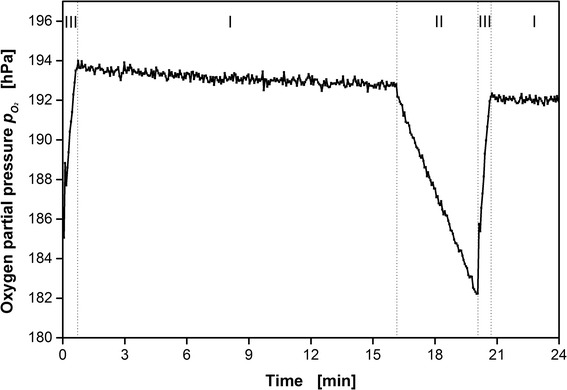
Characteristic segment of a measured single well oxygen partial pressure propagation during cultivation of *H. polymorpha* RB11 pC10-*FMD* (P_FMD_-*GFP*). (I) Phase of standard aeration with air (flow phase), 0.8 vvm, 16 min. (II) Phase of closed inlet and outlet valves and determination of the OTR (stop phase), 0 vvm, 3 min. (III) Phase of elevated aeration with supply air to compensate for the prior interruption of air supply (4.8 vvm, high flow phase). The presented oxygen partial pressure decrease during phase (II) corresponds to an OTR of 21.5 mmol L^-1^ h^-1^. Due to the dynamic equilibrium of oxygen supply and consumption of oxygen, the oxygen partial pressure during phase (I) is reduced and roughly 193 hPa (instead of 209.5 hPa for pure air)

Figure 5 shows a characteristic segment of a measured single well oxygen partial pressure propagation during a μRAMOS cultivation. After phase (I) of standard aeration with air (flow phase, 0.8 vvm, equals 0.64 mL min^-1^ per well at *V*_*L*_ = 800 μL), the valves are closed (II, stop phase, 0 mL min^-1^). Consequently, the oxygen partial pressure decreases due to the continuing respiration of microorganisms. The OTR can be calculated according to Eq.1. To compensate for the interruption of air supply within the stop phase (II), a phase of elevated aeration (III, high flow phase, 3.6 vvm, equals 3.84 mL min^-1^ per well at *V*_*L*_ = 800 μL) is applied. According to the RAMOS measurement principle, these mentioned phases are repeated throughout the entire cultivation. Within this example, an OTR value can be determined every 20 min. If higher sensitivity is required for cultured biological systems with low respiration rates, the duration of phase (II) and (III) (and possibly of phase (I)) can be prolonged. The presented oxygen partial pressure decrease in Fig. 5 during phase (II) corresponds to an OTR of 21.5 mmol L^-1^ h^-1^.

### Utilization of the developed μRAMOS

In the following section, the utilization of the developed μRAMOS is presented by four cultivations. To test the well-to-well deviation, 48 replicates are cultivated initially. To increase the experimental throughput, the replicate number is later reduced to eight and three replicates and lastly singlet cultivations were performed. According to Lattermann et al. [30], the predicted maximum oxygen transfer capacities are 10 mmol/l/h for the plant cell suspension culture and 45 mmol/l/h for the cultivation of *H. polymorpha* and *E. coli* at the applied cultivation conditions. These capacities are smaller compared to a typical stirred tank reactor system. But considering different well geometries and shaking parameters, the resulting increased maximum oxygen transfer capacities can be comparable to stirred tank systems [30–32]. Based on the literature [16, 28, 33, 34], all cultivations of this section are expected to be oxygen unlimited.

### Cultivation of *Escherichia coli*

Starting off with 48 identical cultivations within the MTP, *E. coli* BL21 (DE3) pRotHi-YFP was cultivated in 48 wells and 4 shake flasks simultaneously, to test the comparability between the newly developed MTP-based technology and the well-established RAMOS shake flask system. For the cultivations, parameters were adjusted to allow for sufficient oxygen supply without any oxygen limitation. The duration of the flow and stop phases were adjusted as indicated in the caption of the individual figures.

**Fig. 6:**
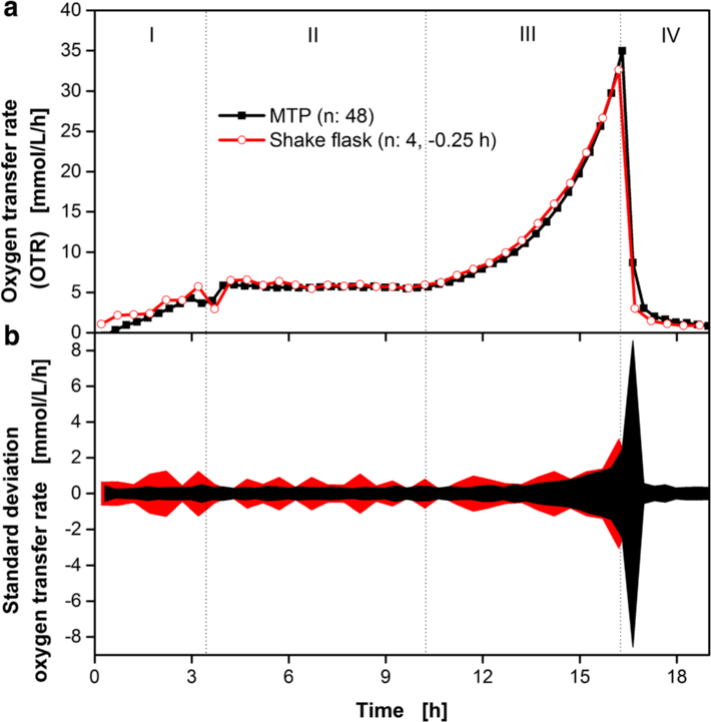
Comparison of *E. coli* BL21 (DE3) pRotHi-YFP cultivations using the newly introduced μRAMOS MTP system (─■─) and the standard RAMOS shake flask system (─○─). Mean values of the measured oxygen transfer rates of 48 wells and 4 shake flask cultivations are shown (**a**). **b** Detailed propagation of corresponding standard deviations of the measured oxygen transfer rates of the 48 wells and 4 shake flask and cultivations. Cultivation medium: Synthetic Wilms-MOPS auto-induction medium with 0.55 g L^-1^ glucose, 2 g L^-1^ lactose and 5 g L^-1^ glycerol. μRAMOS (MTP) cultivation conditions: 48-well Round Well Plate without optodes, *V*_*L*_ = 800 μL, *n* = 1000 rpm, shaking diameter *d*_*0*_ = 3 mm, 37 °C, flow phase + high flow phase: 17 min, stop phase: 3 min., standard RAMOS cultivation conditions: 250 mL RAMOS shake flask, *V*_*L*_ = 10 mL, *n* = 350 rpm, *d*_*0*_ = 50 mm, 37 °C, flow phase + high flow phase: 25 min, stop phase: 5 min

Figure 6 shows the averaged OTR values of the 48 wells and 4 shake flasks. Due to minor temperature differences between the two different cultivation chambers, the shake flask cultivations were slightly shifted for -0.25 h. Based on the obtained online OTR signals, the cultivations can be divided into four characteristic phases (I-IV). These phases have been described in detail by Rahmen et al. [17]. During phase (I), growth on glucose was observed. The depletion of glucose is indicated by a short drop of the OTR at roughly 4 h. Thereafter, the simultaneous consumption of glycerol and lactose as well as product formation (YFP) occurred at a constant OTR of about 6.5 mmol L^−1^ h^−1^ (II). Due to the depletion of lactose, this phase ended after 10 h. The residual glycerol was consumed within the next 6.5 h (III). After the depletion of all carbon sources, the end of the cultivation is indicated by a steep decrease in the OTR (IV) after approximately 16 h. Both mean values of 48-well and 4 shake flask cultivations are in excellent agreement. Small details like the short intermediate decrease of OTR after glucose depletion after roughly 4 h are visible for shake flasks and the wells of the MTP.

Figure 6b shows the corresponding standard deviations of the averaged OTR values of the 48 well and four shake flask cultivations. Until roughly 15 h, the standard deviation of the MTP cultivations was slightly smaller at approximately +/- 0.5 mmol L^-1^ h^-1^ compared to the shake flask cultivations (approximately +/- 1 mmol L^-1^ h^-1^). However, after roughly 15 h, this deviation increased significantly up to +/- 8 mmol L^-1^ h^-1^ and exceeded the corresponding shake flask standard deviation. During this time, the OTR dropped significantly due to the depletion of all carbon sources. Therefore, slight temporal spreads of the 48 cultivations led to different time points of carbon source depletion and, hence, a greater standard deviation of the averaged 48 OTR values at this time. A possible reason for this divergent behavior could be the superior temporal resolution of the MTP measurement. Due to the shorter stop phase and flow phase of the MTP, the measurement rate was increased from two to three OTR values per hour compared to the shake flask. Therefore, the observed temporal spread of the shake flask cultivations appeared less distinct, resulting in smaller standard deviations of the OTR values during this time.

### Cultivation of parsley plant cell suspension culture

**Fig. 7:**
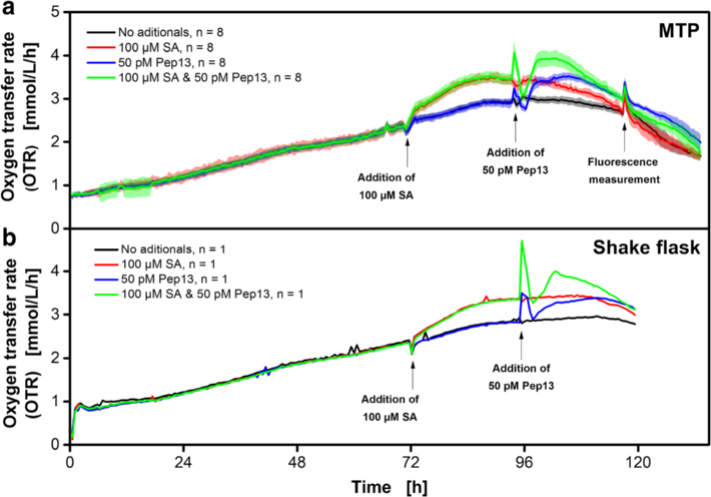
Respiration activity of parsley cell suspension cultures in μRAMOS (MTP) and RAMOS (shake flask) treated with salicylic acid (SA) and a 13 amino-acid defense elicitor of *Phytophthora sojae* (Pep13). Arrows indicate the addition of 100 μM salicylic acid (SA, 72 h) and of a 13 amino-acid defense elicitor of *Phytophthora sojae* (Pep13, 96 h). Shadows in (**a**) symbolize standard deviations of eight cultivations. (**﻿b**) Single shake flask cultivations. Cultivation medium: Gamborg B5 medium with 20 g L^-1^ sucrose. Cultivation conditions μRAMOS (**a**) 48-well Round Well Plate without optodes, *V*_*L*_ = 2000 μL, *n* = 800 rpm, shaking diameter *d*_*0*_ = 3 mm, 25 °C, flow phase + high flow phase: 20 min, stop phase: 10 min, standard RAMOS cultivation conditions (**b**) 250 mL shake flask, *V*_*L*_ = 50 mL, *n* = 180 rpm, *d*_*0*_ = 50 mm, 25 °C, flow phase + high flow phase: 20 min, stop phase: 10 min

Cell suspension cultures of plants can also be cultured in the μRAMOS device. A promising application for parsley (*Petroselinum crispum*) suspension cell cultures is the screening of compounds for an alternative plant protection strategy based on defense priming [34, 35]. The term refers to an alerted state of the cell that enables plants to activate immune and abiotic stress responses faster and stronger than unprimed cells. This is mostly associated with disease resistance and abiotic stress tolerance. [36–38]. Exploiting plant defense priming may help reducing fungicide application [38] thus contributing to sustainable, eco-friendly disease and pest management in the field. Schilling et al. [34] introduced a low-throughput screening system in shake flasks based on the oxygen consumption of parsley suspension cells. They reported an increase in OTR after the addition of a given defense priming compound. The μRAMOS device enables a transfer of the described screening approach to MTPs and, thus, a switch from low to high throughput. Figure 7 shows the accordance of the parsley screening system in an MTP (a) and shake flasks (b). The stronger increase in OTR after addition of the defense priming compound salicylic acid (SA) was compared to the reference and turned out not to differ in MTP and shake flasks. The addition of the defense elicitor Pep13, a biotic stress signal, resulted in a biphasic increase in OTR. The first increase was short but more pronounced and the second increase was more sustained. This increase in oxygen consumption occurred due to the formation of hydrogen peroxide [34]. The response of the primed cultures was more pronounced compared to the non-primed but elicited cultures. These characteristics were found likewise in MTP and shake flasks. These results demonstrate that the resolution of the μRAMOS device is suited for monitoring oxygen consumption of slow-growing parsley suspension cell cultures in small scale.

### Cultivation of *Hansenula polymorpha*

**Fig. 8:**
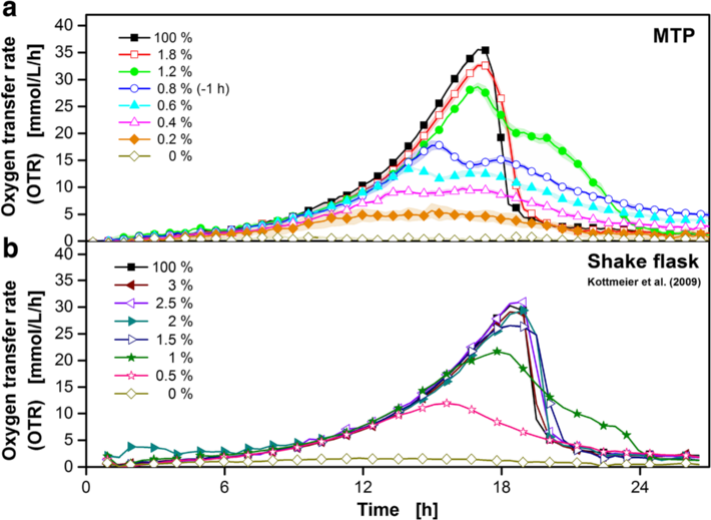
μRAMOS (**a**) and RAMOS (**b**) cultivation of *H. polymorpha* RB11 pC10-*FMD* (P_FMD_-*GFP*) under magnesium limitation. **b** republished data of Kottmeier et al. (2010) [33]. The percentages of magnesium are normalized to the original medium containing 3.0 g L^-1^ MgSO_4_ · 7H_2_O as 100 %. Mean values and corresponding standard deviations (colored shadows) of triple cultivations are shown in (**a**). Single cultivations are shown in (**b**). For clarity, only every second data point over time is indicated by the corresponding symbol in all curves. The cultivations containing 0.8 % magnesium were shifted for -1 h due to possible inoculation variances. Cultivation medium: Synthetic Syn-6-MES medium with 10 g L^-1^ glycerol. μRAMOS cultivation conditions (**a**): 48-well Round Well Plate without optodes, *V*_*L*_ = 800 μL, *n* = 1000 rpm, *d*_*0*_ = 3 mm, 30 °C, flow phase + high flow phase: 16 min, stop phase: 4 min., standard RAMOS cultivation conditions (**b**): 250 mL RAMOS shake flask, *V*_*L*_ = 10 mL, *n* = 300 rpm, *d*_*0*_ = 50 mm, 30 °C, flow phase + high flow phase: 20 min, stop phase: 10 min

Figure 8 shows the μRAMOS (a) and RAMOS (b) cultivation of *H. polymorpha* RB11 pC10-*FMD* (P_FMD_-*GFP*) under different levels of magnesium limitation. The OTR monitoring of eight triplet MTP cultivations (a) and eight singlet shake flask cultivations (b) are shown. The triplet cultivations were averaged and mean values (solid line) and the corresponding standard deviations (colored shadows) are shown. For clarity, only every second data point is indicated by the corresponding symbol in all curves. The results in shake flasks (Fig. 8a) have already been published by Kottmeier et al. [33]. The percentages of magnesium are normalized to the original medium containing 3.0 g L^-1^ MgSO_4_ · 7 H_2_O (100 %). To avoid a different second substrate limitation, Na_2_SO_4_ was added according to the literature [33]. Due to inoculation variances, the three MTP cultivations containing 0.8 % magnesium were shifted for -1 h. In both cultivation vessels, the measurement of the OTR indicates no limiting or inhibiting effect in case of 100 % magnesium. An exponential increase in OTR is observed until glycerol depletion stopped the cultivation after roughly 18 h. The slight temporal shift between the two cultivation vessels of approximately 1 h could be explained by slight inoculation variances and/or slight temperature differences of the two cultivation chambers. In the MTP case (a), the medium containing 1.8 % magnesium (red) shows the first minor but distinct limitation effects: The maximal OTR was slightly smaller and the OTR decrease after roughly 18 h is not as steep compared to the 100 % magnesium cultivation. These trends continue and become more pronounced with decreasing amounts of magnesium. At 0 % magnesium, the OTR remained close to 0 mmol L^-1^ h^-1^. In the case of shake flask (b), the medium containing 1.5 % magnesium showed the first minor, but distinct limitation effects (purple). Similar to the MTP, the maximal OTR value was slightly smaller and the OTR decrease was not as steep compared to the corresponding 100 % magnesium cultivation after roughly 19 h. Also, these characteristics became more pronounced with smaller contents of magnesium applied. The measured OTR curves of the developed μRAMOS system (a) are generally comparable to the published cultivations of Kottmeier et al. (b, [33]). Due to the triplet cultivations (a) instead of eight shake flasks and singlet cultivations (b), additional information on the (in this case excellent) reproducibility is available at a comparable experimental effort. The media consumption was reduced from 10 ml (shake flask) to 0.8 ml (MTP) per cultivation.

**Fig. 9:**
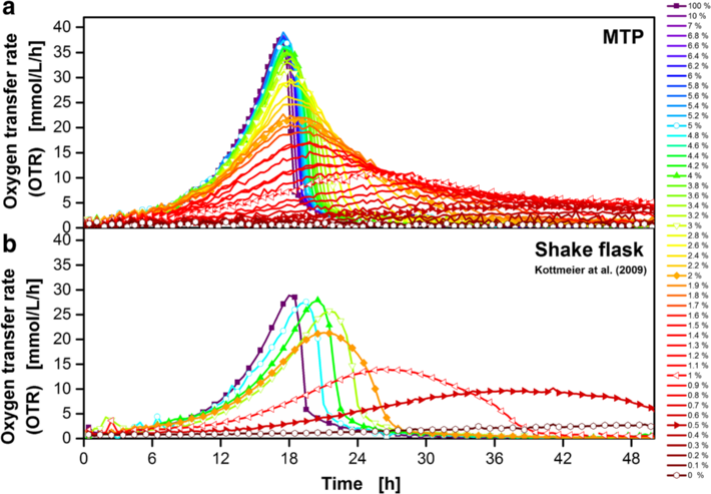
48 different μRAMOS (**a**) and 8 different RAMOS (**b**) cultivations of *H. polymorpha* RB11 pC10-*FMD* (P_FMD_-*GFP*) under potassium limitation. **b** republished data of Kottmeier et al. (2010) [39]. The percentages of potassium are normalized to the original medium containing 1.0 g L^-1^ KH_2_PO_4_ and 3.3 g L^-1^ KCl as 100 %. Single cultivations are shown. For clarity, only the concentrations of the RAMOS cultivations are indicated by a corresponding symbol within the μRAMOS results. Cultivation medium: Synthetic Syn-6-MES medium with 10 g L^-1^ glycerol. μRAMOS cultivation conditions (**a**): 48-well Round Well Plate without optodes, *V*_*L*_ = 800 μL, *n* = 1000 rpm, *d*_*0*_ = 3 mm, 30 °C, flow phase + high flow phase: 16 min, stop phase: 4 min, standard RAMOS cultivation conditions (**b**): 250 mL RAMOS shake flask, *V*_*L*_ = 10 mL, *n* = 300 rpm, *d*_*0*_ = 50 mm, 30 °C, flow phase + high flow phase: 20 min, stop phase: 10 min

Figure 9 shows the comparison of 48 singlet μRAMOS (a) and eight singlet RAMOS (b) cultivations of *H. polymorpha* RB11 pC10-*FMD* (P_FMD_-*GFP*) under different levels of potassium limitation. (b) has already been published in 2010 by Kottmeier et al. [33]. The percentages of potassium are normalized to the original medium containing 1.0 g L^-1^ KH_2_PO_4_ and 3.3 g L^-1^ KCl (100 %). To avoid a different second substrate limitation NH_4_H_2_PO_4_ and NaCl was added respectively according to the literature [33]. For clarity, only every second data point over time of the MTP and shake flask cultivations is indicated by respective symbols. In both cultivation vessels, the measurement of the OTR indicates no limiting or inhibiting effect in the case of 100 % potassium (Fig. 9, (a, b)). Until approximately 18 h, an exponential increase in OTR was observed. During this time, the only carbon source, glycerol, was metabolized. Due to glycerol depletion after roughly 18 h, the OTR decreased abruptly and no further significant respiration activity was observed. Above approximately 5 % potassium, the cultivation was not significantly influenced by a reduced content in the MTP (Fig. 9a). At roughly 4 % potassium, a minor decrease of the maximal OTR value and a slight time shift of the OTR maximum occurred. In the shake flask (Fig. 9b), these trends were partly more distinct. Already starting at 5 % potassium, a time shift of the OTR maximum is clearly visible. The maximal OTR remained on a similar level. This behavior is also shown in the case of 4 % potassium. The first distinct decrease in maximal OTR was observable at 3 % potassium. The MTP cultivation containing 3 % potassium decreased along with its maximal OTR value. Due to the higher cultivation throughput in the MTP, the gradual behavior between a potassium content of 4 and 3 % can be resolved. In both cultivation vessels, the clearly observable trend of decreasing maximal OTR values accompanied by a time shift of this OTR maximum remained comparable. When 0 % potassium was applied, the OTR stayed close to 0 mmol L^-1^ h^-1^ for the monitored timespan in the MTP. In the shake flask, a slightly elevated OTR at roughly 3 mmol L^-1^ h^-1^ after 48 h was observed. In summary, aside from minor differences, both systems deliver similar results of comparable quality with the major benefit of a 6-fold higher cultivation throughput in the MTP. Hence, the resolution of the investigated influence of a potassium limitation is increased 6-fold at a comparable experimental effort. The quality of the obtained μRAMOS OTR signals is sufficient for performing singlet cultivations. Additionally, the media consumption was reduced from 10 ml (shake flask) to 0.8 ml (MTP) resulting in a 12.5-fold decrease per cultivation.

The respective results of a third second substrate limitation (phosphate) of *H. polymorpha* are shown in Additional file 1.

## Conclusions

This paper introduces a newly developed 48-well MTP-based OTR monitoring system called μRAMOS. Initially, the necessary valves and oxygen sensors were implemented into a newly developed microfluidic MTP cover. A comparison of experimental results from the μRAMOS as well as the established shake flask RAMOS system is presented using four examples. The data demonstrate that the new MTP-based system offers a similar quality of online OTR determination. Additionally, 48 identical *E. coli* cultivations showed only minor well-to-well deviations, comparable to the shake flask standard. Different experimental approaches are shown, in which three (Fig. 8) or eight (Fig. 7) replicate cultivations are performed instead of 48 identical cultivations. Thus, important information on the reproducibility could be obtained, combined with an increased throughput. The experimental throughput is maximized in the case of 48 different singlet cultivations (Fig. 9), for which the obtained OTR signal quality was found to be sufficient. This way, the resolution of the investigated influence of a potassium limitation could be increased 6-fold compared to the shake flask RAMOS system. An additional benefit of the new introduced MTP-based μRAMOS system is the 12.5-fold reduction of media consumption per cultivation while applying typical filling volumes of 10 mL in shake flask and 0.8 mL in MTP cultivations.

## Methods

### Microorganisms and cells

In this study, two different microorganisms, one parsley plant suspension cell culture and four general media were applied: *E. coli* BL21 (DE3) pRotHi-YFP on synthetic Wilms-MOPS auto-induction medium, *H. polymorpha* RB11 pC10-*FMD* (P_FMD_-*GFP*) on modified synthetic Syn-6-MES media with different amounts of magnesium, potassium and phosphate and a parsley cell suspension culture on synthetic Gamborg B5 medium [34]*. E. coli* BL21 (DE3) pRotHi-YFP and *H. H. polymorpha* RB11 pC10-*FMD* (P_FMD_-*GFP*) were kindly provided by the Institute for Molecular Enzyme Technology (IMET) and the Institute for Microbiology at the Heinrich-Heine-University Düsseldorf (Germany), respectively.

### Media and cultivation

All pre-cultures of *E. coli* BL21 (DE3) pRotHi-YFP and *H. polymorpha* RB11 pC10-*FMD* (P_FMD_-*GFP*) were carried out in standard 250 mL shake flasks.

For *E. coli* BL21 (DE3) pRotHi-YFP, two pre-cultivations were conducted according to the literature [17, 27, 28]. The first pre-cultivation on terrific broth (TB) medium was inoculated with 1 ml terrific broth (TB) cryocultures (200 g L^−1^ glycerol stocks, stock OD_600_ = 1). The second pre-culture was conducted on Wilms and Reuss medium (henceforth referred as Wilms-MOPS medium [39]) with 20 g L^-1^ glucose, 0.2 M MOPS buffer, and 50 mg L^-1^ sterile filtrated kanamycin. Both pre-cultivations were carried out at *T* = 37 °C, a shaking frequency of *n* = 350 rpm, with a shaking diameter *d*_*0*_ = 5 cm and a filling volume of *V*_*L*_ = 10 mL. For the main cultivation, a modified Wilms-MOPS auto-induction medium containing 0.55 g L^-1^ glucose, 2 g L^-1^ lactose and 5 g L^-1^ glycerol as carbon sources was applied. The pH was not controlled, since a sufficiently buffered medium was applied.

For the pre-cultivation of *H. polymorpha* RB11 pC10-*FMD* (P_FMD_-*GFP*), synthetic Syn-6-MES medium was prepared according to the literature [27, 40]. The pre-cultivation was inoculated with 1 mL Syn-6-MES medium cryoculture (200 g L^−1^ glycerol stocks) and cultivated at *T* = 30 °C. A shaking frequency of *n* = 300 rpm, a shaking diameter *d*_*0*_ = 5 cm and a filling volume *V*_*L*_ = 10 mL were applied. The modified Syn-6-MES media for the main cultures with different amounts of magnesium (Fig. 8), potassium (Fig. 9) and phosphate (Additional file 1) were prepared according to the literature  [33]. It contained among other nutrients 10 g L^−1^ glycerol and 0.2 M MOPS buffer. The pH was not controlled, since a sufficiently buffered medium was applied.

Parsley cells (*Petroselinum crispum*) in suspension were cultivated in Gamborg B5 medium in a 48-well Round Well Plate (MTP-R48-B, m2p labs GmbH, Baesweiler, Germany) covered with a sterile barrier (900371-T, HJ-Bioanalytik GmbH, Erkelenz, Germany). The filling volume was set at *V*_*L*_ = 2000 μL, the shaking frequency was *n =* 800 rpm, with a shaking diameter *d*_*0*_ = 3 mm and *T* = 25 °C. Shake flask cultivations were conducted in 250 mL RAMOS shake flasks as a reference as described by Schilling et al. [34] with *V*_*L*_ *=* 50 mL, *n* = 180 rpm and *d*_*0*_ = 50 mm at *T* = 25 °C. The same batch of suspension cells was used for the cultivation in MTP and shake flasks. Furthermore, measurement phases were identical for MTP and shake flasks for the parley suspension cell culture. Flow phase plus high flow phase was 20 min followed by a stop phase of 10 min.

Stock solutions of 10 mM salicylic acid (SA) and 50 nM Pep13 [41] (W398500, Sigma-Aldrich Chemie GmbH, Munich, Germany) were stored at −20 °C. Both solutions were diluted with distilled water at a ratio of 1:2. MTP and shake flasks were supplemented with 1 mL or 40 μL, respectively.

### Symbols

*d*_*0*_, Shaking diameter [mm]

$\frac{\varDelta {\boldsymbol{p}}_{{\boldsymbol{O}}_2}}{\varDelta \boldsymbol{t}}$, Oxygen partial pressure change over time during stop phases [hPa h^-1^]

*n*, Shaking frequency [rpm]

*R,* Gas constant [hPa mol^-1^ K^-1^]

*T,* Temperature [K]

*V*_*G*_, Sealed gas volume [L]

*V*_*L*_, Liquid filling volume [mL]

## Additional file

Additional file 1: μRAMOS MTP cultivation of *H. polymorpha* RB11 pC10-*FMD* (P_FMD_-*GFP*) under phosphate limitation. Corresponding results from shake flask can be found in Kottmeier et al. (2010) for comparison [33]. The percentages of phosphate are normalized to the original medium containing 1.0 g L-1 KH_2_PO_4_ as 100 %. Mean values (data points) and corresponding standard deviations (colored shadows) of triple cultivations are shown. For clarity, every third data point over time is indicated by the corresponding symbol. Cultivation medium: Synthetic Syn-6-MES medium with 10 g L-1 glycerol, cultivation conditions: 48well Round Well Plate without optodes, *V*_*L*_ = 800 μL, *n* = 1000 rpm, *d*_*0*_ = 3 mm, 30 °C, flow phase + high flow phase: 16 min, stop phase: 4 min. (TIF 561 kb)

## Abbreviations

*E. coli*: *Escherichia coli* BL21 (DE3) pRotHi-YFP
GFP: Green fluorescent protein
*H. polymorpha*: *Hansenula polymorpha* RB11 pC10-*FMD* (P_FMD_-*GFP*)
MTP: Microtiter plate
OD: Optical density
OTR: Oxygen transfer rate [mol L^-1^ h^-1^]
Pep13: Cell wall peptide of *Phythophthora sojae*, used as a defense elicitor
RAMOS: Respiration activity monitoring system
SA: Salicylic acid
YFP: Yellow fluorescent protein
