# Supplementary figures and images for: Respiration activity monitoring system for any individual well of a 48-well microtiter plate
Source: J Biol Eng. 2016 Oct 27;10:14. doi: 10.1186/s13036-016-0034-3 (PMC5081973; doi:10.1186/s13036-016-0034-3)

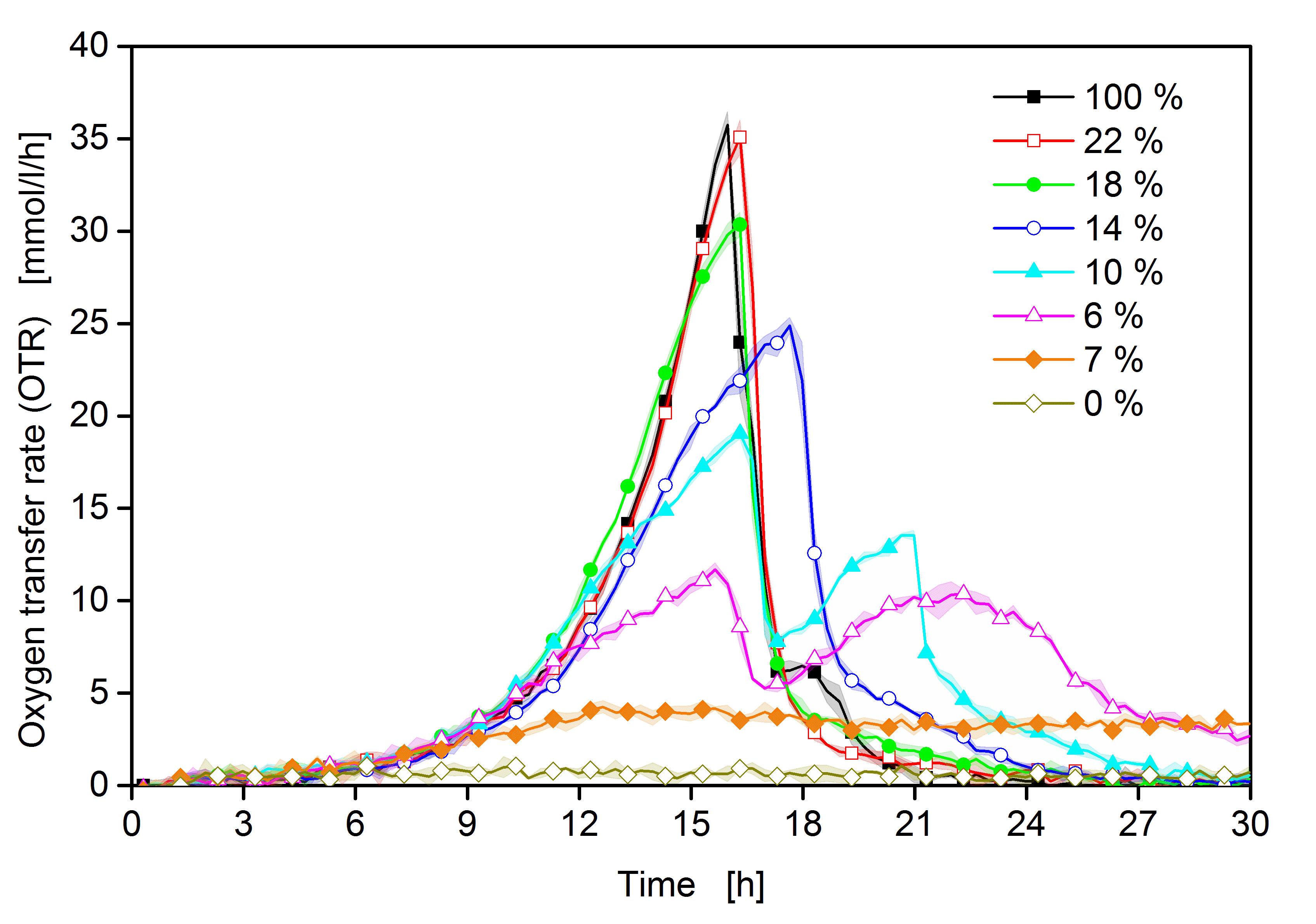

Supplement: Additional file 1: — μRAMOS MTP cultivation of H. polymorpha RB11 pC10-FMD (PFMD-GFP) under phosphate limitation. Corresponding results from shake flask can be found in Kottmeier et al. (2010) for comparison [33]. The percentages of phosphate are normalized to the original medium containing 1.0 g L-1 KH2PO4 as 100 %. Mean values (data points) and corresponding standard deviations (colored shadows) of triple cultivations are shown. For clarity, every third data point over time is indicated by the corresponding symbol. Cultivation medium: Synthetic Syn-6-MES medium with 10 g L-1 glycerol, cultivation conditions: 48well Round Well Plate without optodes, V L = 800 μL, n = 1000 rpm, d 0 = 3 mm, 30 °C, flow phase + high flow phase: 16 min, stop phase: 4 min. (TIF 561 kb) [file 13036_2016_34_MOESM1_ESM.tif]
